# Calomel and Ulcerative Colitis in Septic Conditions

**Published:** 1907-09-14

**Authors:** 


### Points in Treatment.

### CALOMEL AND ULCERATIVE COLITIS IN SEPTIC CONDITIONS.

The way in which septic conditions and ulcerative colitis are connected pathologically as cause and effect is not known for certain, but the fact that they are connected in some way or other is beyond doubt. It is not at all uncommon to find acute colitis, and even ulcerative colitis, at autopsies upon persons suffering from such lesions as fungating endocarditis, abdominal suppuration which has lasted days and weeks rather than hours and days, pneumococcal septicæmia, pyelonephritis, and so on.

It is also generally believed that calomel in repeated doses may itself produce acute colitis, and even ulceration; and with calomel may be included several other drastic purgatives.

Putting these two points together—namely, the tendency of repeated purgatives to inflame the colon, and the tendency of persons suffering from extensive sepsis to develop colitis—it is not at all surprising that not a few patients who have suffered from abdominal or other sepsis, and who have been treated upon the principle that purgation was the main indication, have very acute inflammation and ulceration of their colons when post-mortem examination is made. The colitis is so much more frequent when calomel has been given than when it has not that it seems certain that the administration of repeated doses of this purge is the actual cause of the bowel lesion in these cases. There can be little doubt that the ulcerative colitis in such cases accelerates the end, for patients with this and no other lesion are very much below par.

It by no means follows that calomel or some other purgative is contra-indicated in septic states; on the contrary, in suitable doses, and with suitable modifications in each case, it undoubtedly does good; the point we wish to make is that the purgation may itself be a source of danger. The calomel treatment is possibly used by surgeons more than it is by physicians, and for this very reason it is necessary to lay stress upon its dangers; for surgeons do not regularly see the post-mortem results, and the ulcerative colitis is a thing which can only be fully appreciated by actually seeing it at autopsy.

It is by no means uncommon, if signs of sepsis or sapræmia develop after an abdominal operation, or after labour, for example, to see calomel given at once; if the bowels are not freely opened as the result the calomel is apt to be repeated, or some other potent purgative may be given in its stead. In cases of peritonitis there may be no action of the bowels in spite of all these, on account of paralytic distension of the bowel, or, on the other hand, severe diarrhœa may set in. In either case the patient is very apt to have ulcerative colitis, which will be found at autopsy if the original trouble is so severe as to cause death.

It may be urged that the ulceration of the large intestine might have occurred in these cases as the direct result of the peritoneal or other sepsis, even if purgatives had not been given. This would be very difficult to disprove in any particular single case; but when two series of similar conditions are taken, in one of which there was extensive use of purgatives such as calomel, and in the other no such strenuous efforts to get the bowels open, the proportion of cases in which ulcerative colitis occurs is very much greater in the former group than it is in the latter. The heroic use of calomel, in other words, undoubtedly renders the patient much more liable to the bowel lesion, and this is the point which we wish to emphasise here. Powerful purgatives often repeated may cause acute colitis in cases of Bright's disease in a similar way.

It is very difficult, no doubt, to say how many patients who recover after operations for general peritonitis owe their recovery in part at least to the free administration of laxatives or purgatives; it is equally difficult to say for certain whether any similar patients who are not treated with purgatives, and who die, might have recovered if purgatives had been given. The matter is largely a question of opinion; but there is now a growing opinion that purgatives after these operations are best administered in very strict moderation only. The liability to actual colitis is an additional argument in favour of such moderation.

The following case is one of a series that occurred in the days when calomel was freely used. A woman aged 33 was confined on September 22; she had had five children previously without trouble, but on this occasion, though she had been in perfect health up to full term, she had a very bad labour. There was much hæmorrhage from placenta-prævia, twins were born dead, and afterwards the patient became blanched, and collapsed from further loss of blood. She did well until September 26, but then showed signs of sepsis; the breasts were tender, the lochia suppressed, and the temperature rose to 101° F. Calomel was given to open the bowels, but the trouble grew worse instead of better. The treatment consisted of the usual local measures together with antistreptococcic serum hypodermically and calomel by the mouth. The patient gradually sank from septic exhaustion, and died on October 3; the bowels had not been opened more than twice a day, or at most three times. At the autopsy, the only abnormal organs were the uterus and the colon. On opening the uterus, a condition of acute endometritis was seen, involving the entire interior both of the body of the uterus and of its cervical canal. In the ascending colon there was deep purple blotching of the mucosa and sub-mucosa, with thick granular exudate upon the internal surface, and shallow but extensive ulceration; the condition looked quite recent—more recent than the labour. There can be little doubt that the ulcerative colitis arose from the extensive use of calomel in a patient whose powers of resistance were already lowered from the existence of severe sepsis elsewhere. One cannot say that such a patient would have recovered if calomel had not been given; but one feels quite certain that, had she recovered, the ulcerated state of her colon would have given her great trouble afterwards.

